# Transcription of Toll-Like Receptors 2, 3, 4 and 9, FoxP3 and Th17 Cytokines in a Susceptible Experimental Model of Canine *Leishmania infantum* Infection

**DOI:** 10.1371/journal.pone.0140325

**Published:** 2015-10-14

**Authors:** Shazia Hosein, Alhelí Rodríguez-Cortés, Damer P. Blake, Karin Allenspach, Jordi Alberola, Laia Solano-Gallego

**Affiliations:** 1 Royal Veterinary College, Pathology and Pathogen Biology, University of London, Hawkshead Lane, North Mymms, AL9 7TA, United Kingdom; 2 Universitat Autònoma de Barcelona, LeishLAB-SAF, Departament de Farmacologia de Terapèutica i de Toxicologia, 08193 Cerdanyola del Vallès, Barcelona, Spain; 3 Royal Veterinary College, Clinical Sciences and Services, University of London, Hawkshead Lane, North Mymms, AL9 7TA, United Kingdom; 4 Universitat Autònoma de Barcelona, Facultat de Veterinaria, 08193 Cerdanyola del Vallès, Barcelona, Spain; Instituto Oswaldo Cruz, Fiocruz, BRAZIL

## Abstract

Canine leishmaniosis (CanL) due to *Leishmania infantum* is a chronic zoonotic systemic disease resulting from complex interactions between protozoa and the canine immune system. Toll-like receptors (TLRs) are essential components of the innate immune system and facilitate the early detection of many infections. However, the role of TLRs in CanL remains unknown and information describing TLR transcription during infection is extremely scarce. The aim of this research project was to investigate the impact of *L*. *infantum* infection on canine TLR transcription using a susceptible model. The objectives of this study were to evaluate transcription of TLRs 2, 3, 4 and 9 by means of quantitative reverse transcription polymerase chain reaction (qRT-PCR) in skin, spleen, lymph node and liver in the presence or absence of experimental *L*. *infantum* infection in Beagle dogs. These findings were compared with clinical and serological data, parasite densities in infected tissues and transcription of IL-17, IL-22 and FoxP3 in different tissues in non-infected dogs (n = 10), and at six months (n = 24) and 15 months (n = 7) post infection. Results revealed significant down regulation of transcription with disease progression in lymph node samples for TLR3, TLR4, TLR9, IL-17, IL-22 and FoxP3. In spleen samples, significant down regulation of transcription was seen in TLR4 and IL-22 when both infected groups were compared with controls. In liver samples, down regulation of transcription was evident with disease progression for IL-22. In the skin, upregulation was seen only for TLR9 and FoxP3 in the early stages of infection. Subtle changes or down regulation in TLR transcription, Th17 cytokines and FoxP3 are indicative of the silent establishment of infection that *Leishmania* is renowned for. These observations provide new insights about TLR transcription, Th17 cytokines and Foxp3 in the liver, spleen, lymph node and skin in CanL and highlight possible markers of disease susceptibility in this model.

## Introduction

Leishmaniases are diseases caused by more than 20 species of protozoa within the genus *Leishmania*, following transmission by the bite of phlebotomine sand-flies. Both human and canine leishmaniosis (CanL) are classed as neglected diseases and are endemic in the Mediterranean Basin, the Middle East and tropical and sub-tropical regions of the world [1].

*Leishmania infantum* is the species most commonly associated with canine infections [2]. This infection in dogs can manifest as chronic subclinical infection, self-limiting disease or severe illness and is largely prevalent in the Mediterranean Basin and Brazil [3]. In dogs, the main effector mechanism involved in protective immunity is the activation of macrophages by IFN-γ and TNF-α to eliminate intracellular amastigotes via the L-arginine nitric oxide pathway [4]. Disease development is often correlated with increasing parasite burdens together with a strong but inefficient humoral response [3].

In recent years, there has been plenty of evidence defining immune responses of specific organs/tissues during *Leishmania* infection. However, much of this information derives from murine models [5] and it is well established that findings from murine studies are inconsistent when translated to the canine or human systems [6].

The innate immune responses associated with parasitic infections have been reported previously [7] and in more recent times toll-like receptors (TLRs) have been shown to play an important role in leishmaniosis [8]. TLRs distinguish pathogen-associated molecular patterns (PAMPs) derived from viruses, pathogenic bacteria, fungi and parasitic protozoa. TLRs are type 1 integral membrane glycoproteins of tri-modular structure [9]. TLRs, with other innate receptors, play a vital role in innate immune responses in addition to shaping adaptive immunity [9]. While most studies have focused on bacterial and fungal pathogens, recent studies have demonstrated that TLRs, in particular TLRs 2, 3, 4 and 9 may play a major role in recognition of protozoan pathogens such as *Leishmania* [8]. These TLRs appear to up-regulate and activate pro-inflammatory responses in infected macrophages resulting in killing of the parasite. These studies are based mainly on investigations into *L*. *major* infections in the mouse model [8]. *Leishmania major* results in cutaneous leishmaniasis (CL) and the immune responses associated with CL and CanL differ greatly. In contrast, there is a very limited body of data available in the characteristics of innate immunity in dogs after infection with *L*. *infantum*.

Th17 cells play a vital role in the protection of surfaces against many extracellular and fungal pathogens but have also been implicated in the mediation of severe immune pathologies. IL-17 is involved in the recruitment, migration and activation of neutrophils. [10]. IL-22 is also produced by Th17 cells and to a lesser extent by natural killer and Th1 cells [10], and is particularly involved in immunity at epithelial and mucosal surfaces [11]. IL-17 and IL-22 are inflammatory cytokines that play a protective role against intracellular parasites such as *Leishmania* [12]. However, the role of IL-17 and IL-22 during *Leishmania* infection remains controversial and poorly defined [13,14]. On the other hand, T regulatory lymphocytes (Tregs) have an important role in suppression of host immunity in murine [15,16] and human leishmaniasis [17,18] and probably also in CanL [19,20]. Tregs are characterised by the expression of CD4, CD25, and the highly conserved transcription factor Forkhead box P3 (Foxp3) serving a pivotal role in stabilising their regulatory properties [21]. However, Th17 cytokines [22] and the transcription factor FoxP3 have been scarcely studied in CanL [19,20] and especially not in the visceral organs and require further investigations. In this study we aimed to evaluate the transcription of TLR2, TLR3, TLR4, TLR9, as well as the cytokines IL-17 and IL-22 and transcription factor FoxP3 in lymph node, skin, liver and spleen from *L*. *infantum* experimentally infected and uninfected dogs. Previous studies have reported cytokine profiles associated with this disease [23,24], but there is a very limited amount of data describing TLR transcription and FoxP3 in the visceral organs or skin of dogs. In particular, no studies to date have reported TLR3 transcription in these organs and to our knowledge IL-17 and IL-22 have not been reported in CanL.

## Materials and Methods

### Ethical statement

All procedures were approved by the local Ethical Review Committee of Zoetis, Olot, Spain (formerly known as Fort Dodge Veterinaria S.A.) in compliance with national (*Real Decreto* 1201/2005) and European Union regulations (European Directive 86/609/CE) for projects using animals for research purposes. The project license number assigned by the Ethics Committee that approved the study was number 132 and the permit number allocated by the Spanish Authorities was number 3303.

### Study groups

#### Clinically healthy, non-infected control dogs

Liver, spleen, skin and popliteal lymph node samples were obtained within fifteen minutes of euthanasia from 10 clinically healthy Beagle dogs at Pfizer Ltd. (Sandwich, Kent, UK). Dogs were euthanized by intravenous overdose of barbiturate. All dogs were male and between 36 to 72 months of age at the time of necropsy and were previously used as control subjects in other studies. Tissue samples were cut into 5mm^3^ pieces using sterile scissors and immediately transferred to cryo-tubes containing RNA-later (Ambion, UK) and stored immediately according to manufacturer’s instructions and indefinitely at -80°C until further use.

#### Experimentally infected dogs

Experimentally infected animals comprised of thirty one six-month old, intact female beagles from which we obtained end-point tissue samples to perform the present study.

Dogs were housed in pens of six animals (13.2m^2^/pen) or seven animals (15.2m^2^/pen). Dogs had direct contact with pen mates and with neighbouring pens and daily exercise in the facility corridors. There was environmental enrichment in the form of toys (ropes, Kong toys stuffed with treats and chew bones) and the inside of each cage had an elevated platform where dogs could jump up onto or hide below. All dogs had previously received routine vaccinations and were additionally treated with anthelmintic drugs. None of the dogs had detectable levels of anti-*Leishmania* antibodies in serum determined by enzyme linked immune sorbent assay (ELISA) [25]. Serum samples were also obtained for IgG, IgA and protein A ELISAs. ELISA’s were performed as described previously [25] with some modifications and employed sonicated crude total *Leishmania* antigen. Briefly, microtitre plates were coated with 2 μg of *L*. *infantum* antigen per well. Polyclonal anti-dog immunoglobulin IgG, IgA (Bethyl Laboratories, Montgomery, TX, USA) and Protein A (ProtA) (ImmunoPure® Recomb® Protein A, Pierce Rockford, IL, USA) were all conjugated to horseradish peroxidase and were used individually as secondary antibodies [26,27]. Protein A reacts with the Fc-region of Ig from many species including canines [28]. Absorbance values were read at 492 nm in an automatic microELISA reader (Anthos Labtec Instruments) and reactions were expressed in ELISA units (EU), which was in relation to a known positive serum sample used as a calibrator and set at 100 EU. This calibrator serum was always the same and included in all plates. Sera from 32 dogs living in a non-endemic region for CanL were used to set up a cut-off for IgG and IgA. This was established at 7.6 EU for IgG and 60 EU for IgA and 14 EU for Protein A. Any plate with an inter-assay variation > 10% was discarded [27].

### Experimental infection and follow-up

Dogs were experimentally infected with a total dose of 2 x 10^8^ *L*. *infantum* amastigotes (MCAN/ES/92/BCN-83/MON-1, kindly provided by Dr Montserrat Gallego, Universitat de Barcelona) via the intravenous route (i.v.). Briefly, *L*. *infantum* obtained from a naturally infected dog which had not received any treatment was passaged through hamsters to retain virulence. Amastigotes were isolated from spleens of infected hamsters and used immediately for experimental infection. The removed spleen was homogenized in Schneider’s *Drosophila* medium (Sigma, St Louis, MO, USA) supplemented with 20% foetal calf serum (Gibco, Paisley, UK) and 25 μg/ML gentamicin (Sigma) and centrifuged at 500g for 10 minutes at 4°C. The supernatant was washed three times and re-suspended in 0.9% physiological saline solution. The number of viable parasites was determined after staining with crystal violet. Dogs were infected by i.v. injection of 2 x 10^8^ amastigotes in 1mL of saline solution. Following infection, clinico-pathological findings associated with CanL were recorded monthly and scored as detailed elsewhere [29]. Scores were added to give an overall clinico-pathological record for each dog. Specifically, each animal underwent a complete physical examination monthly by the same veterinarian and blood was collected for complete blood count and biochemistry analysis including renal and hepatic function. Clinical findings including weight loss, cutaneous lesions and lymphadenomegaly were scored from one to three and biochemical and haematological findings were scored by assigning one point to each parameter with an abnormal value. To minimise suffering of the dogs during infection, clinical end points for withdrawal were established based on clinical signs of the disease and the veterinarian notified if any such observations were made. Such notifications included severe skin lesions, severe lameness, debilitating diarrhoea, neurological signs or significant bleeding from any orifice.

Twenty four of the experimentally infected dogs were euthanized at 6 months post infection (Group 1) and seven were euthanized at 15 months post infection (Group 2). There were no deaths during the study period in any of the two groups. At necropsy, popliteal lymph node, spleen, liver and ear skin samples were collected from all dogs and samples were snap-frozen in liquid nitrogen. Dogs were anaesthetized with buprenorphine (dose: 6 mcg/kg) plus dexmedetomidine hydrochloride (dose: 12.5 mg/kg) or azepromacine (dose: 100 mg/kg) administered intramuscularly. Approximately 30 minutes after anaesthesia they were euthanized with an overdose (>200 mg/kg) of sodium pentobarbital (Dolethal®, Vetoquinol) intravenously.

### Determination of parasite densities in infected tissues

DNA extractions from tissues were performed using a High Pure PCR Template Preparation Kit (Roche, San Francisco, USA) according to manufacturers’ instructions. *Leishmania* DNA in tissues was detected by quantitative real-time polymerase chain reaction (qRT-PCR) as previously described [30]. Briefly, PCR mastermix was made using Taqman® Universal Master Mix II (Applied Biosystems, USA) with *Leishmania* kinetoplast DNA specific primers designed by Francino et al [30] (Leish1 forward primer 5’-AACTTTTCTGGTCCTCCGGGTAG’3’, Leish2 reverse 5’-ACCCCCAGTTTCCCGCC-3’) and MGBProbe 5’-AAAATGGGTGCAGAAAT-3’ (Applied Biosystems, UK). Assays were performed with a 25 μL final volume with 5 μL of sample DNA. The standard curve was established from *Leishmania* DNA extracted from 1 × 10^7^ parasites; 5 μL of serial dilutions, ranging from 10^3^ to 10^−3^ parasites, was placed into reaction tubes. The cycling conditions were; an incubation step at 50°C for 2 minutes, a denaturation step at 95°C for 10 minutes followed by 40 cycles at 95°C for 15 s and 60°C for 1 minute. All PCR analyses were performed in a Step One Plus Real Time PCR System (Applied Biosystems Laboratories, Foster City, CA, USA). Each standard, negative control and sample were analysed in triplicate.

### RNA extraction, RNA integrity and cDNA generation

Tissue samples stored at -80°C in RNAlater (Ambion, Paisley, UK) or snap-frozen in liquid nitrogen were thawed on ice and RNA extracted using an Ambion RiboPure Kit (Ambion) according to the manufacturer’s instructions with a DNase digestion step included to remove contaminating genomic DNA using TurboDNase (Ambion, Paisley, UK). RNA concentration was determined by nanodrop using the Nanodrop ND-2000 spectrophotometer (ThermoScientific, Wilmington, DE, USA) and samples were diluted in nuclease free water appropriately to give a final concentration of 40 ng/μL per sample. The RNA integrity was determined using an Agilent 2100 Bioanalyzer (Agilent Technologies, Waldbronn, Germany). All samples included in this study had an RNA Integrity Number (RIN) value in excess of six.

cDNA was generated using an Oligo(dT)_15_ primer (Promega, Southampton, UK) and IM-prom-II Reverse Transcriptase enzyme (Promega, Southampton, UK) according to the manufacturer’s instructions. cDNA was aliquoted and stored at -20°C.

### Reference gene and target gene selection

Reference and target genes were as shown in Tables 1 and 2. The three most appropriate reference genes for each tissue were subsequently determined using geNorm normalization (Biogazelle, Belgium). Selected genes were used to calculate the geometric mean from which absolute quantification of target genes were performed.

**Table 1:** Primer sequences for selected reference genes.

Gene name (abbreviation)	Primer sequence 5’– 3’	Product size (base pairs)	Anneal. temp (°C)	GenBank accession number	Tissues employed	Reference
**Hypoxanthine phosphoribosyl-transferase 1 (HPRT1)**	F: CACTGGGAAAACAATGCAGA	123	56	AY_283372	Liver	[31]
	R: ACAAAGTCAGGTTTATAGCCAACA				Lymph node	
					Spleen	
**Hydroxymethyl-bilane synthase (HMBS)**	F: TCACCATCGGAGCCATCT	112	57	XM_546491	Liver	[31]
	R: GTTCCCACCACGCTCTTCT				Lymph node	
**Beta 2 –microglobulin (B2M)**	F: ACGGAAAGGAGATGAAAGCA	99	56	XM_535458	Lymph node	[31]
	R: CCTGCTCATTGGGAGTGAA				Spleen	
**Succinate dehydrogenase complex subunit A (SDHA)**	F: GCCTTGGATCTCTTGATGGA	92	56	XM_535807	Liver	[31]
	R: TTCTTGGCTCTTATGCGATG				Skin	
**Glyceraldehyde– 3—phosphate dehydrogenase (GAPDH)**	F: GGAGAAAGCTGCCAAATATG	193	55	NM_001003142	Spleen	[32]
	R: ACCAGGAAATGAGCTTGACA				Skin	
**HIRA interacting protein 5 (HIRP5)**	F: GGCTTTGAAGATGGCATTGT	127	56	XM_850340	Skin	[33]
	R: CACCCTCCACCTCTGGAATA					

**Table 2:** Primer sequences for target genes of interest.

Target gene(abbreviation)	Primer sequence 5’– 3’	Product size (base pairs)	Annealing temp (°C)	GenBank accession number	Reference
**Toll-like receptor 2 (TLR2)**	F: AGTGGCCAGAAAAGCTGAAA	263	54.3	NM_001005264	[34]
	R: ATCCAGTTGCTCCTTCGAGA				
**Toll-like receptor 3 (TLR3)**	F:GTCCAATTTCATTAAGGCCAAG	129	54	XM_540020.2	This study
	R:TGTCACTTGCTCAGTCTCCTTT				
**Toll-like receptor 4 (TLR4)**	F: CAGCATTCCAGTTTGAAGCA	140	54.3	AB080363	This study
	R: AAGACTTCGAGGCTGACCAA				
**Toll-like receptor 9 (TLR9)**	F: GCCTGGAGTACCTGCTCTTG	128	55	NM_001002998.1	This study
	R: GTTACGGGCATGGTCACAG				
**Interleukin 17A (IL-17A)**	F: CCGATCTACCTCACCTTGGA	166	60	NM_001165878.1	[35]
	R: TCGCAGAACCAGGATCTCTT				
**Interleukin 22 (IL-22)**	F: TCCAGCAGCCCTATATCACC	254	60	XM_538274.2	[35]
	R: TTGGCTTAGCTTGTTGCTGA				
**Forkhead box P3 (FoxP3)**	F: GGCTCCTGCTGTATCGTAGC	179	55	NM_001168461.1	This study
	R: CGCATGTTGTGGAATTTGAA				

### Creation of PCR standard serial dilutions for qRT-PCR

PCR amplicons representing each test assay were purified and ligated into the cloning vector pGEM®-TEasy (Promega, Southampton, UK) and then transformed using OneShot® Top10 Chemically Competent *Escherichia coli* (Invitrogen) according to the manufacturer’s instructions. Plasmid extraction was performed using the PureYield Plasmid Miniprep System (Promega, Southampton, UK) according to the manufacturer’s instructions and used in conventional PCR with the primers used to generate the original amplicon to confirm the presence of the insert. PCR mastermix was created by combining nuclease free water (NFW), Hi-Spec additive (Bioline, Taunton, MA, USA), Immobuffer (Bioline, Taunton, MA, USA), MgCl_2_ (4.5 mM), dNTP mix (100 mM), specific primer mix (800 nM) and Immolase Taq (0.02 U/μlL (Bioline, Taunton, MA, USA). 5 μL diluted cDNA was then added to 20 μL of mastermix to give a final volume of 25 μL. Negative controls were performed by the addition of nuclease free water instead of cDNA. PCR cycling conditions included enzyme activation (95°C) for seven min followed by 35 cycles of denaturation at 94°C for 40 s, annealing at specific primer temperature for 30 s and elongation at 72°C for 60 s. Finally, an extension step was performed at 72°C for seven min.

Plasmids shown to have the specific insert were sent for Sanger sequencing (Source BioScience LifeSciences, UK) and subsequently aligned with the original gene sequence using CLC Workbench version 6 (CLCBio, Denmark). The DNA concentration of eluted plasmids with the required insert were determined by nanodrop (Nanodrop DN-2000, ThermoScientific, Wilmington, DE, USA) then diluted with nuclease free water to a concentration of 1 x 10^7^ copies/uL calculated using the plasmid nucleotide size and Avogadros constant, and a 10-fold serial dilution series was created with a lowest concentration of 1 x 10^1^ copies/uL.

### qRT-PCR

Target cDNA numbers were quantified using real time PCR using Ssofast Evergreen Supermix (Bio-Rad, CA, USA), target specific primers (Tables 1 and 2) and Cycler programs on the CFX96 Real time detection system (Bio-Rad laboratories, CA, USA) were created according to the manufacturer’s recommendations for optimized cycling conditions for RT-PCR. This comprised of an enzyme activation step at 95°C for 30 s (hot start), followed by 40 cycles of denaturation at 95°C for 5 s, annealing at specific primer annealing temperature (55–60°C, Tables 1 and 2) for 10 seconds and a melting curve analysis of 65–95°C with a plate read every 0.5°C at the end of the 40 cycles. Standard curves (ranging from 10^7^ to 10^1^ copies per μL) were generated from plasmids of known DNA concentration to allow absolute quantification of target gene transcription. Each reaction was carried out in triplicate and the reaction efficacy was determined for each gene using ten-fold dilutions (10^7^ molecules/μl to 10^1^ molecules/μl). Nuclease free water was used in place of DNA in triplicate as the non-template control. Absolute gene transcription was quantified by averaging triplicate quantities of each sample for all test genes, following normalisation of the expression ratio of each using the geometric mean of three reference genes.

### Statistical analysis

SPSS (IBM, USA) statistical software (Version 21) was used for statistical analyses. Statistical differences between groups were judged using the Kruskal-Wallis test with the Mann-Whitney U- test used for post hoc comparison. Correlations between parasite densities in infected tissues and clinical score data and all other parameters were performed using Spearman’s rank-order correlation. Mann-Whitney U test was used to judge differences between groups, for clinical data, serology and parasite density in infected tissues. R statistical software (Version 3.1.1) was used to construct heat maps for transcription level. Type I error rate, Alpha (α), was set at 5%, and Bonferroni correction was used to evaluate significance for post-hoc comparison.

## Results

### Clinical data, serology and parasite density in infected tissues

Control dogs from the UK were deemed clinically healthy and were not included in comparisons. Mann-Whitney U test revealed a significant increase (*p*<0.001) in clinical scores between infected groups. Median (interquartile range [IQR]); Group 1 = 2.87 [0–0.73] and Group 2 = 22.55 [15.88–28.73].

Dogs from Group 1 presented with few or no clinical signs or clinico-pathological abnormalities with a mean clinical score of 2.67 and were seronegative or low seropositive. Dogs from Group 2 presented clinical signs such as weight loss, alopecia, and exfoliative dermatitis, lymphadenomegaly in addition to clinico-pathological abnormalities with a mean clinical score of 21.58 and had high IgG and IgA antibody levels. Clinical scores significantly increased between infected groups (*p*<0.001).

IgG and IgA serum levels measured by ELISA were both higher in Group 2 (300 [296.04–311.75] and 119.38, [74.0–201.97], respectively, when compared with Group 1 (medians = 145.09 [23.02–281.29] and 79.21 [20.72–181.68]). This increase was statistically significant for IgG (*p*<0.001) but not for IgA (*p* = 0.796). Mann-Whitney U test revealed a significant increase (*p*<0.001) in protein A between the infected groups (Group 1 median = 95.64 [10.37–174.71], Group 2 median = 1350.00 [600–1500.00]).

Parasite densities in infected tissues were expressed as the number of parasites per milligram of tissue and not for the whole tissue as organs were not weighed. In the skin, the parasite density increased significantly between the two time points of infection [Median = 95.85 (1.60–2455.10) and 1651 (3.50–47616.80) respectively, *p* = 0.038]. An increase in parasites/mg tissue was also seen in the lymph node and a decrease noted in the liver, but these differences were not significant. On the contrary, the parasite density in spleen samples was found to significantly decrease with disease progression (*p* = 0.022). These findings are summarized in Table 3.

**Table 3:** Median (range) and p-values for the parasite densities in infected tissues (parasites per mg/tissue) in both infected groups.

Tissue	Group 1 (6 months post infection)	Group 2 (15 months post infection)	Group 1 vs Group 2
**Spleen**	21005.95 (0–110083.30)	2365.10 (17.10–39808.00)	p = 0.022*
**Liver**	29450.70 (60.90–140482)	10668.30 (37.50–73515.10)	p = 0.199
**Lymph node**	897.55 (0–22410.10)	1595 (16.60–5774.20)	p = 0.695
**Skin**	95.85 (1.60–2455.10)	1651.70 (3.50–47616.80)	p = 0.038*

(*) shows significant differences (**p = <0.05**).

### Absolute quantification of target gene transcription

#### Liver

Transcription of IL-22 was down regulated in the presence of *L*. *infantum* when the controls were compared with each infected group. TLR2 transcription was up regulated with disease progression and no differences were seen among groups in other cytokines, FoxP3 and TLRs (Table 4).

**Table 4:** Median (range) and p-values for the transcription of target genes within liver samples.

Genes (Liver)	Controls	Group 1 (6 months post infection)	Group 2 (15 months post infection)	Controls vs Group 1	Controls vs Group 2	Group 1 vs Group 2
**TLR2**	96.58 (84.42–1392.73)	550.77 (128.43–903.51)	366.63 (201.42–892.36)	p = 0.001*	p = 0.007*	p = 0.764
**TLR3**	239.34 (159.73–3256.20)	298.42 (95.98–831.85)	214.97 (103.31–288.47)	p = 0.809	p = 0.962	p = 0.695
**TLR4**	162.66 (118.09–2348.42)	203.00 (47.76–400.73)	132.04 (90.40–303.30)	p = 0.148	p = 0.315	p = 0.417
**TLR9**	556.32 (336.05–7636.49)	1387.00 (192.72–7405.74)	497.47 (149.37–956.36)	p = 0.196	p = 0.109	p = 0.502
**IL-17**	0.58 (0.27–6.98)	0.49 (0.08–1.99)	1.80 (1.63–17.4)	p = 0.377	p = 0.571	p = 0.060
**IL-22**	6.63 (3.36–76.40)	0.46 (0.12–5.73)	2.83 (2.10–5.05)	p<0.001*	p = 0.033	p<0.001*
**FoxP3**	2.25 (2.05–32.54)	3.59 (0.81–11.61)	2.04 (1.35–36.79)	p = 0.480	p = 0.232	p = 0.563

Significant Bonferroni corrected p-values are highlighted bold (p< = α/3).

(*) p-value<0.016.

#### Lymph node

Transcription was significantly down regulated for all genes of interest (TLR2, TLR3, TLR4, TLR9, IL-17, IL-22 and FoxP3) with the exception of TLR9, when Group 1 were compared with the controls. Comparing Group 2 and the controls, down regulation was observed for transcription of all genes of interest with the exception of TLR2 (Table 5).

**Table 5:** Median (range) and p-values for the transcription of target genes within lymph node samples.

Genes (Lymph node)	Controls	Group 1 (6 months post infection)	Group 2 (15 months post infection)	Controls vs Group 1	Controls vs Group 2	Group 1 vs Group 2
**TLR2**	1221.20 (591.02–2049.55)	567.61 (266.89–4039.30)	1148.49 (946.55–1644.48)	p = 0.001*	p = 0.740	p = 0.001*
**TLR3**	2287.15 (1091.26–5313.42)	310.27 (136.98–2233.89)	146.77 (100.96–202.02)	p<0.001*	p<0.001*	p = 0.001*
**TLR4**	1838.31 (1212.52–3401.61)	349.76 (130.66–1490.22)	284.16 (230.33–388.74)	p<0.001*	p <0.001*	p = 0.872
**TLR9**	1565.95 (987.70–3183.26)	2012.24 (665.90–7213.90)	651.89 (341.12–943.30)	p = 0.287	p<0.001*	p <0.001*
**IL-17**	19.38 (0–55.43)	3.45 (0–22.95)	5.96 (0.71–7.01)	p = 0.002*	p = 0.005*	p = 0.729
**IL-22**	67.88 (13.74–1440.20)	4.95 (1.18–352.62)	1.87 (0.92–3.35)	p<0.001*	p <0.001*	p = 0.002*
**FoxP3**	1029.74 (324.94–2005.54)	197.26 (45.63–1248.18)	35.05 (20.14–55.11)	p<0.001*	p<0.001*	p<0.001*

Significant Bonferroni corrected p-values are highlighted bold (p< = α/3).

(*)p-value<0.016.

#### Spleen

Significant differences in transcription were observed in the spleen samples for TLR4, TLR9 and IL-22. Comparing Group 1 with the controls, transcription of TLR4 and IL-22 was down regulated, whereas TLR9 was up regulated. Comparing Group 2 with the controls, transcription of TLR4 and IL-22 was also down regulated. No differences were seen among groups in IL-17, FoxP3 and TLRs (Table 6).

**Table 6:** Median (range) and p-values for the transcription of target genes within spleen samples.

Genes(Spleen)	Controls	Group 1 (6 months post infection)	Group 2 (15 months post infection)	Controls vs Group1	Controls vs Group 2	Group 1 vs Group 2
**TLR2**	3007.40 (1798.94–3287.07)	2383.58 (1550.86–4654.14)	2544.15 (1568.13–5424.72)	p = 0.118	p = 0.417	p = 0.627
**TLR3**	1028.55 (517.35–2312.03)	741.91 (157.04–2191.08)	367.96 (147.59–1120.88)	p = 0.515	p = 0.088	p = 0.085
**TLR4**	3765.41 (2733.20–4570.21)	1157.07 (483.38–2924.33)	920.75 (445.67–1293.38)	p = 0.002*	p = 0.005*	p = 0.182
**TLR9**	980.30 (560.20–1222.08)	6866.13 (778.07–119451.01)	2316.45 (395.51–26120.09)	p<0.001*	p = 0.364	p = 0.167
**IL-17**	1.46 (0.53–6.57)	2.27 (0.66–12.49)	1.84 (1.05–3.19)	p = 0.393	p = 0.905	p = 0.273
**IL-22**	17.67 (11.68–35.42)	4.33 (0.94–22.27)	3.04 (0.18–20.26)	p<0.001*	p = 0.002*	p = 0.661
**FoxP3**	43.73 (20.67–85.44)	62.78 (7.51–210.02)	39.94 (18.33–101.60)	p = 0.222	p = 0.758	p = 0.139

Significant Bonferroni corrected p-values are highlighted bold (p< = α/3).

(*)p-value<0.016.

#### Skin

Transcription of TLR3 and TLR9 was down regulated in the later stages of infection (comparing Groups 1 and 2). Transcription of TLR2 and TLR9, together with FoxP3, were all up regulated in the earlier stages of infection when compared with the controls. No differences were seen among groups in cytokines and other TLRs (Table 7).

**Table 7:** Median (range) and p-values for the transcription of target genes within skin samples.

Genes (Skin)	Controls	Group 1 (6 months post infection)	Group 2 (15 months post infection)	Controls vs Group 1	Controls vs Group 2	Group 1 vs Group 2
**TLR2**	67.93 (35.42–100.44)	201.43 (107.29–316.82)	130.71 (129.19–198.86)	p = 0.042	p = 0.171	p = 0.462
**TLR3**	277.62 (155.65–399.59)	351.49(99.57–802.59)	125.43 (60.34–125.60)	p = 0.728	p = 0.257	p = 0.038
**TLR4**	157.39 (120.51–194.28)	172.54 (118.81–267.37)	103.71 (102.90–323.95)	p = 0.262	p = 0.762	p = 0.940
**TLR9**	307.64 (214.71–400.57)	1152.08 (509.62–1625.92)	174.57 (73.13–229.63)	p = 0.007*	p = 1.000	p = 0.044
**IL-17**	0.43 (0.23–0.64)	0.98 (0.31–9.06)	2.70 (0.98–4.12)	p = 0.218	p = 0.200	p = 0.282
**IL-22**	9.57(2.15–16.99)	5.01 (1.91–9.02)	2.51 (0.28–5.92)	p = 0.924	p = 0.171	p = 0.093
**FoxP3**	1.07 (0.82–1.33)	11.06 (1.53–21.54)	6.50 (1.73–8.93)	*p = 0.019^*	*p = 0.019^*	p = 0.550

Significant Bonferroni corrected p-values are highlighted bold (p< = α/3).

(*)p-value<0.016.

(^) indicates marginal significance.

### Parasite densities in infected tissues correlated with all other parameters

#### Six months post infection

**Fig 1:**
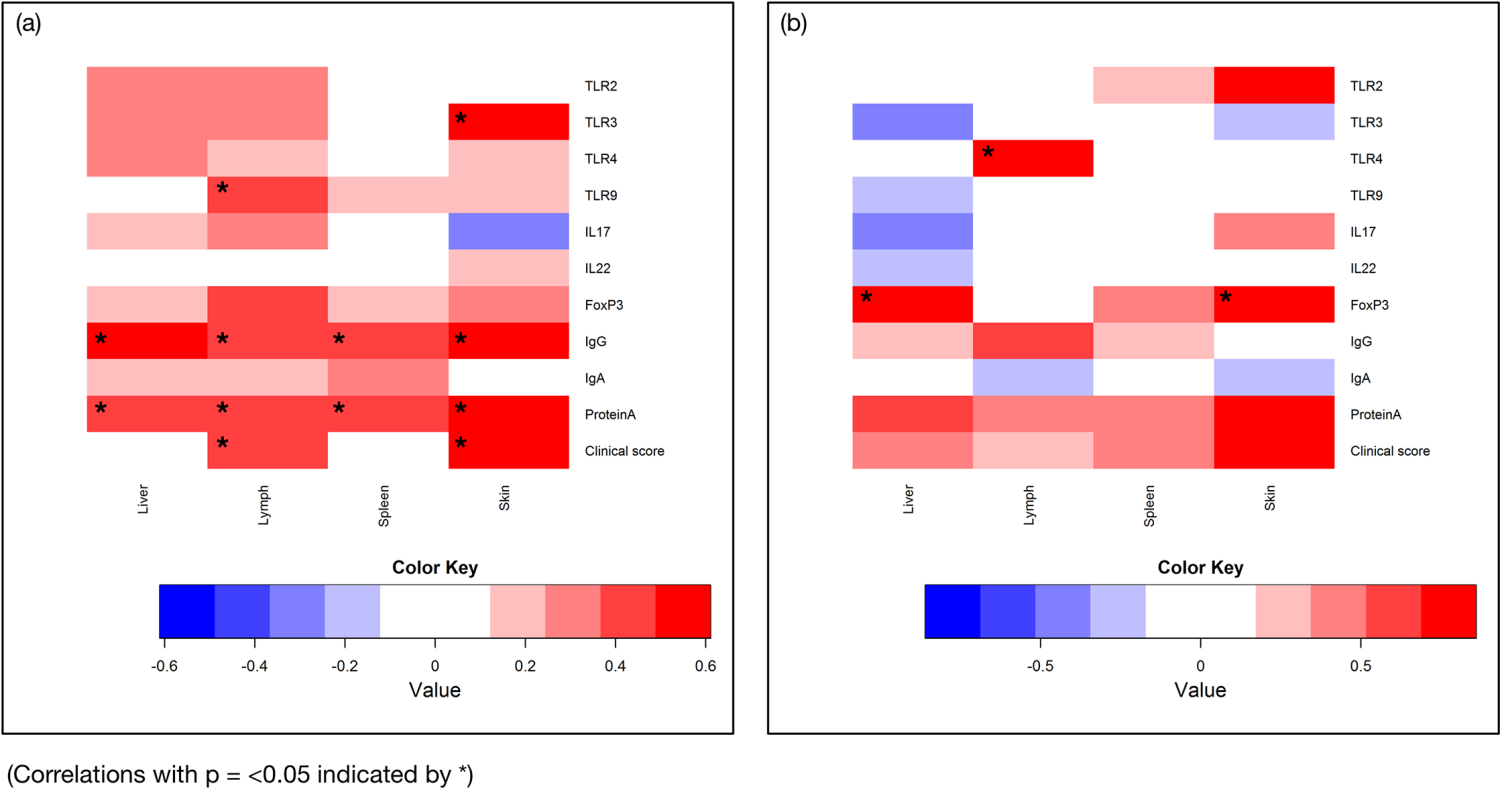
Heat maps illustrating the positive (red) and negative (blue) correlation values between parasite densities at six months post infection (a) and fifteen months post infection (b) in liver, lymph node, spleen and skin with transcription from genes of interest, serological parameters and clinical scores.

In the liver, there was moderate positive correlation between parasite density and IgG serum levels (*r*_*s*_ = 0.504, *p* = 0.012), and serum protein A (*r*_*s*_ = 0.469, *p* = 0.048). In the lymph node, moderate correlation with parasite density was revealed for TLR9 transcription (*r*_*s*_ = 0.427, *p* = 0.037), IgG serum levels (*r*_*s*_ = 0.420, *p* = 0.041), serum protein A (*r*_*s*_ = 0.460, *p* = 0.024) and clinical score (*r*_*s*_ = 0.437, *p* = 0.037). In the spleen, serum IgG (*r*_*s*_ = 0.462, *p* = 0.023) and serum protein A (*r*_*s*_ = 0.470, *p* = 0.020) were also positively correlated with the parasite density. Lastly, in the skin, moderate to strong correlation was seen for TLR3 transcription (*r*_*s*_ = 0.556, *p* = 0.005), serum IgG (*r*_*s*_ = 0.560, *p* = 0.004), serum protein A (*r*_*s*_ = 0.611, *p* = 0.002) and clinical score (*r*_*s*_ = 0.598, *p* = 0.002) when correlated with parasite density in infected skin. These correlations are all illustrated in the heat map shown in Fig 1A together with all other correlations with parasite densities in infected tissues at six months post infection.

#### Fifteen months post infection

At 15 months post infection, strong positive correlation was revealed when parasite densities were correlated with FoxP3 transcription in both liver and skin (*r*_*s*_ = 0.821, *p* = 0.023 and *r*_*s*_ = 0.860, *p* = 0.019, respectively) and also TLR4 transcription in the lymph node (*r*_*s*_ = 0.821, *p* = 0.023). These correlations are all illustrated in the heat map shown in Fig 1B together with all other correlations with parasite densities in infected tissues at 15 months post infection.

### Clinical score correlation with all other parameters

#### Six months post infection

**Fig 2:**
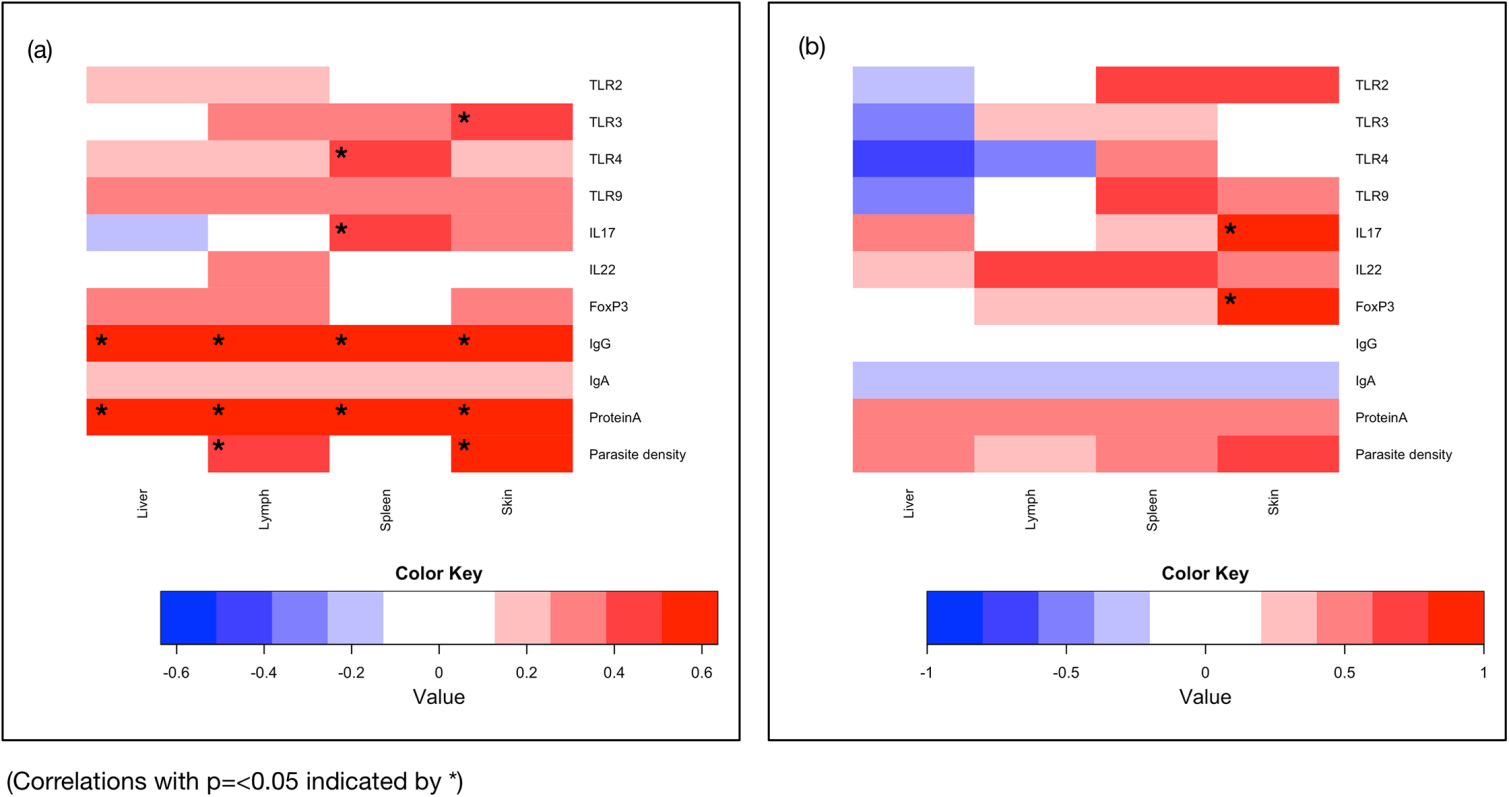
Heat maps illustrating the positive (red) and negative (blue) correlation values between clinical score at six months post infection (a) and fifteen months post infection (b) in liver, lymph node, spleen and skin with transcription from genes of interest, serological parameters and parasite densities.

There was strong positive correlation between clinical score and IgG serum levels (*r*_*s*_ = 0.623, *p* = 0.001), and serum Protein A (*r*_*s*_ = 0.636, *p* = 0.001). In the lymph node, there was a moderate correlation between clinical score and parasite density (*r*_*s*_ = 0.437, *p* = 0.037). In the spleen, TLR4 transcription (*r*_*s*_ = 0.483, *p* = 0.017) and IL-17 transcription (*r*_*s*_ = 0.499, *p* = 0.013) were also positively correlated with the parasite density. Lastly, in the skin, moderate to strong correlation was seen for TLR3 transcription (*r*_*s*_ = 0.447, *p* = 0.029), and parasite density (*r*_*s*_ = 0.598, *p* = 0.002) when correlated with clinical score. These correlations are all illustrated in the heat map shown in Fig 2A together with all other correlations with clinical score at six months post infection.

#### Fifteen months post infection

At 15 months post infection, strong positive correlation was revealed in the skin for both IL-17 and FoxP3 transcription (*r*_*s*_ = 1, *p* <0.001 in both instances). These correlations are all illustrated in the heat map shown in Fig 2B together with all other correlations with clinical score at 15 months post infection.

## Discussion

The impact of *Leishmania* sp. infection on toll-like receptors has mainly been studied in murine and human models [36] [37] [38] [39] [40]. To date, there is a limited amount of information about the role that these receptors play in CanL [19,41,42]. This study aimed to investigate the association between *L*. *infantum* infection and transcription of TLRs 2, 3, 4 and 9 in the liver, spleen, lymph node and skin of dogs.

Within all four tissues studied, the largest differences were seen in the lymph node. Lymph nodes are believed to be the first pertinent lymphoid tissues to be affected following *Leishmania* dissemination from skin macrophages [24] in natural infection. Comparing controls to Group 1, transcription of all TLRs and cytokines observed was down regulated significantly with the exception of TLR9. TLR2 has been associated with a protective role in a number of murine models of leishmaniosis. In a *L*. *major* murine model, the absence of TLR2 led to an increased number of cutaneous lesions [43] and TLRs 2 and 3 were involved in the phagocytosis of *L*. *donovani* parasites [44]. Most recently, it was reported that the macrophages derived from peripheral blood from *Leishmania* infected dogs showed a decrease in TLR2 compared with healthy non-infected dogs [45] in agreement with our findings.

There was a general trend of down regulation in the lymph nodes of these dogs as time progressed for all target genes with the exception of TLR2. TLR2 transcription was down regulated in the early stages of infection and then increased between Groups 1 and 2. This correlates with the silent establishment of infection that *Leishmania* is renowned for. A silent phase was reported by Belkaid and colleagues in mice infected with *L*. *major* over a period of 4–5 weeks before the development of lesions [46]. The silent phase in our study was further supported by the down regulation of IL-22 transcription. The silent establishment was also reported in a dog cytokine study where a low proportion of experimentally infected dogs expressed specific cytokines in the first eight months of infection coupled with parasite dispersion, but without clinical signs of disease [47]. The authors suggested that this was the parasite ‘silent establishment’ by avoiding adverse host-cell mediated immunological reactions [47]. Ehrchen and colleagues demonstrated that mice lacking peripheral lymph nodes were more susceptible to *L*. *major* infection and elicited a Th2 response [48]. This finding illustrated that lymph nodes are crucial for assisting in the induction of a protective Th1 response. This could further explain the progressive onset of disease in the animals in this study.

The down regulation of TLR3 in the lymph node has also been associated with the establishment of disease, as observed in the present study. TLR3 is reported to be needed for nitric oxide production and parasite phagocytosis [44], therefore a down regulation in this TLR would favour disease progression. Using a hamster model, Ives and colleagues demonstrated that metastasizing *L*. *guyanensis* parasites have a high *Leishmania* RNA virus–1(LRV1) burden which is recognized by the host TLR3 to induce pro-inflammatory cytokines and chemokines and exacerbation of disease [49]. The absence of TLR4 in a knockout murine *L*. *major* model resulted in increased cutaneous lesions [36] and treatment with TLR4 and TLR9 agonists lessened disease progression [38]. In the lymph node of the present study, TLR4 transcription was down regulated in both infected groups compared with controls and TLR9 transcription was down-regulated with disease progression. TLR9 deficient mice exhibited a deficit in lymph node expansion following *L*. *major* infection and increased susceptibility [50]. The down regulation of TLR3, TLR4, TLR9, IL-17 and IL-22 transcription seen between the infected groups in our study are indicative of disease establishment.

In the skin, no significant changes were seen for TLR2, TLR3, TLR4, IL-17 or IL-22 between any of the three groups studied. This supports the silent establishment of infection as reported earlier. Only TLR9 was significantly up regulated in the early stages of infection and marginal significance was revealed for FoxP3 when both Groups 1 and 2 were compared with controls. The trend for down regulation of the TLRs with disease progression seen in this tissue is suggestive of an inhibitory role where the parasite may be facilitating the onset of disease by reducing or limiting the transcription of these TLRs which would otherwise play a protective role. Tuon and colleagues looked at the TLR9 expression in patients with CL caused by *L*. *braziliensis*. The percentage of cells that expressed TLR9 was significantly higher in the skin of CL patients compared to normal skin but TLR9 expression was down-regulated in keratinocytes of infected patients [39]. The exact reason for this difference is unclear but the authors speculated that higher TLR9 in granulomas could possibly have been a mechanism of parasite control and the avoidance of more generalized and widespread dermatitis [39]. In another human CL study, Tolouei and colleagues recently reported that the mean relative gene expression and membrane expression of TLR2 and TLR4 in the macrophages of patients with the healing form of cutaneous lesions were significantly higher than patients with the non-healing form of lesion [51]. These studies suggest that there is a distinct role for both these TLRs in the outcome of CL lesion as a result of *L*. *major* infection [51].

Recently in a canine study, increased frequency and expression of TLR9 was associated with a lower parasite load in the jejunum of *L*. *infantum* infected dogs, whereas the colon showed a higher parasite load along with an increased frequency and expression of TLR2 [19]. These results are similar to our findings and might suggest active innate immune and pro-inflammatory responses due to the presence of high *Leishmania* parasite densities in infected tissues. There are even fewer reports documenting TLR expression or transcription in the liver and spleen, especially in dogs. A recent study in mice has shown that infection with *L*. *infantum* resulted in the increased transcription of TLR2 and TLR4 and associated cytokines. At all time points observed, animals had a higher TLR2 mRNA transcription in the spleen compared with non-infected controls [52]. This increased transcription could have been due to an influx of inflammatory cells into the spleen, in particular at the beginning of the infection, and a decrease seen at the chronic stage of infection possibly due to partial control of the infection [52]. In contrast, in the present study, TLR4 transcription was down regulated with infection and disease progression as well as other TLRs and cytokines. It must be taken into consideration that murine and canine *L*. *infantum* experimental and natural infections are very different [6,53] as shown with the results of this study. Recently, Melo and colleagues reported increased TLR2 and TLR4 expression in spleen which is opposite to our findings in this tissue [42]. These differences could be due to the fact that the dogs in the Melo study were naturally infected and disease progression would be slower and subject to different immunological responses to the experimentally infected animals in the present study. In a human study, Kumar *et al* reported significantly higher levels of mRNA encoding both TLR2 and TLR4 in pre-treatment splenic aspirate samples but no changes to TLR9 between these groups during *L*. *donovani* infection [54].

To the best knowledge of the authors, this is the first study of Th17 cytokines in several tissues in dogs experimentally infected with *L*. *infantum*. In addition, very limited data is available about these cytokines in other canine diseases [35]. Both of these Th17 cytokines decreased with disease progression in the present study. A reduction of these cytokines was previously reported in dogs with canine inflammatory bowel disease [35]. As with many other cytokines, there have been conflicting reports, suggesting both a protective and pathological role for these Th17 cytokines in both human and rodent leishmaniases.

In an experimental leishmaniasis model, Th17 cells were associated with tissue destruction. Mice deficient in IL-17 suffered smaller lesions with lower neutrophil infiltration compared to wild type mice, which showed no reduction in parasite load with increased IL-17 [13]. Studies with murine models of *L*. *braziliensis* [55] and *L*. *panamensis* [56] infections showed that lesional healing was associated with elevated IL-17 and IFN-γ whereas a report of human muco-cutaneous leishmaniasis (ML) reported that IL-17 might be involved in ML pathogenesis rather than cure [57].

Conversely, there have been a few studies in recent years that have suggested that Th17 cytokines may be associated with protection against leishmaniasis. Ghosh et al demonstrated that administration of recombinant IL-17 and IL-23 caused a significant suppression of organ parasite burden in mice with a marked generation of IFN-γ and nitric oxide. The effects of this therapy were much faster for IL-17 compared with IL-23 [58]. In a human kala-azar study, it was reported that both IL-17 and IL-22 were associated with a protective role [59]. Along with signature Th1 cytokines, IL-17 and IL-22 were found to have complementary roles in protection against kala-azar as it was postulated that a defect in Th17 induction could increase the risk of kala-azar [59]. In the present study, progressive down regulation of IL-22 transcription in all tissues and of IL-17 transcription in the lymph node suggests that these cytokines might have a protective role for CanL. In a murine model, *L*. *infantum* was found to induce IL-17A production which promoted the control of parasite replication by strengthening Th1 responses [12]. More studies investigating the role of these Th17 cell cytokines in CanL would be required to determine the precise role they play in this infection.

The transcription factor FoxP3 was also measured in this study. T regulatory cells are believed to act to counteract inflammatory immune responses, often limiting tissue damage. The absence of these cells has been linked to a number of autoimmune conditions [60]. In human leishmaniasis, elevated intra-lesional FoxP3 and IL-10 were associated with unresponsiveness to treatment during *L*. *amazonensis* infection [61]. However, recently, Silva and colleagues reported an increase in IL-10 production by Treg cells in the spleens of dogs naturally infected with *L*. *infantum* and suggested that this could be due to persistent immune activation triggered by the infection process [20]. In our study, no significant changes were seen in the spleen for this transcription factor.

In the present study, we report a significant reduction in FoxP3 transcription in the lymph node with disease progression. The parasite burden in the lymph node was also increased between the two infected groups and taken together these findings suggest that FoxP3 is required to control the infection. A decreasing trend in FoxP3 transcription between infected groups together with increasing parasite density between these groups in the skin also support the notion that FoxP3 might be associated with a protective role in this infection. Figueiredo and colleagues recently found that *L*. *infantum* infection in dogs increased the expression of TGF-β, IFN-γ, TNF-α and FoxP3 in the jejunum and colon of dogs [19]. In the present study, FoxP3 transcription significantly increased in the skin between controls and sub-clinical (Group 1) animals, whereas in the lymph node, FoxP3 transcription significantly decreased with disease progression suggesting that transcription can vary depending on the tissue sampled. Menendez-Souza and colleagues conversely reported that lower levels of FoxP3 were observed in the skin of sick dogs compared with sub-clinical dogs and this was negatively correlated with clinical progression [22]. There is very little information about the role of FoxP3 in CanL but in murine studies with *Leishmania donovani*, it has been reported that FoxP3 mediates *L*. *donovani* persistence in the liver of mice [62]. It is possible that that role of FoxP3 in leishmaniasis differs depending on not only the causal *Leishmania* species but also on the tissue studied and host species as demonstrated in this study.

It is well established that serum IgG levels measured using either crude *Leishmania* antigens or recombinant proteins correlate strongly with disease severity in human leishmaniasis [63] and in CanL [64,65]. In the present study, the total IgG increased significantly with disease progression. A marked increase in this immunoglobulin as seen here has been well documented before [27,29,66]. IgA was also increased with disease progression as previously observed [29] but in our study but this was not significant. Earlier studies have also indicated that IgA is increased in sick infected dogs compared with healthy infected or non-infected dogs [26,67]. High concentrations of *Leishmania*-specific IgA have additionally been reported in human VL patients [68] and tegumentary leishmaniasis [69].

Parasite burden in the different tissues varied in this study. Although not ideal, intravenous inoculation is believed to be the best route to induce clinical illness because it allows the quick spread of live parasites to different organs [6,29,70]. Following i.v. challenge, in comparison to the visceral organs, the skin is one of the last tissues to be invaded by the parasite. This has been well documented in a hamster model [71] and also in dogs [72–74] and has been reiterated in this study. Parasite density in infected skin increased progressively with disease progression by comparison of Groups 1 and 2 as expected, even though each group was only sampled once. Repeated sampling of each individual animal would have been expected to yield more detail. It was recently reported that a high parasite number in skin is a good predictor of infectiousness [75]. This progression would be expected in natural infections where the disease would be transmitted by sand-fly bite. Thus, a higher load in the skin in late infection would be advantageous to promote transmission onto a new host. The reduced number of parasites per/mg of tissue in the liver and spleen seen in this study is likely to be due to the fact that this was not a longitudinal investigation and readings between animals varied. However, obtaining samples of visceral organs from the same animal at different time points would have been very difficult and not cost effective.

There are several limitations of the present study. Unfortunately, samples were only available from endpoint tissue samples at six and 15 months post infection. It was therefore not possible to evaluate innate responses at the time of experimental infection or correlate features of these responses with disease development through a prospective longitudinal study. However, evaluating innate immune responses at six and 15 months post infection is of relevance. Although classically the fate of infection appears to depend on the adaptive immune response, it is important to take into account that over time the parasites would continue to replicate and chronically infect new cells and hence have an impact on innate immune responses as seen in this study and interact with adaptive immune responses. In addition, it must be noted that the sample sizes between groups were not evenly matched and that control dogs were of different ages and gender to the infected dogs. Although not ideal, these samples provide a base line to compare with the infected animals. The controls were from a non-endemic region and were of the same breed. Due to the limited data that is presently available in *in vivo* models of this kind, the findings from this study are crucial to assist in the further understanding of the immune responses associated with CanL.

The cytokines and TLR profiles in different compartments where *L*. *infantum* replicates appear to have a varied effect on local parasite control. The data described in this study sheds more light on the complex immune responses that develop within the dog to *L*. *infantum* infection as the parasite progresses through its life cycle. This is the first report to highlight fluctuations in the transcription of TLRs, IL-17, IL-22 and FoxP3 in the liver, spleen, lymph node and skin that associate with *L*. *infantum* infection in dogs. Future studies are still needed to clarify these findings and perhaps put them into the context of a natural infection. The precise immune responses that are capable of controlling this infection must be identified in order to progress with development of preventative approaches and/or treatment regimes for this disease.
